# The Administration of 100% Oxygen and Respiratory Drive in Very Preterm Infants at Birth

**DOI:** 10.1371/journal.pone.0076898

**Published:** 2013-10-18

**Authors:** Jeroen J. van Vonderen, Nadia E. Narayen, Frans J. Walther, Melissa L. Siew, Peter G. Davis, Stuart B. Hooper, Arjan B. te Pas

**Affiliations:** 1 Department of Pediatrics, Division of Neonatology, Leiden University Medical Center, Leiden, The Netherlands; 2 The Ritchie Centre/Monash Institute for Medical Research, Clayton, Victoria, Australia; 3 Department of Newborn Research, Royal Women's Hospital, Melbourne, Victoria, Australia; Icahn School of Medicine at Mount Sinai, United States of America

## Abstract

**Aim:**

To retrospectively investigate the changes of SpO_2_ and respiratory drive in preterm infants at birth after administration of 100% oxygen.

**Methods:**

Respiratory parameters, FiO_2_ and oximetry of infants <32 weeks gestation before and after receiving FiO_2_ 1.0 were reviewed during continuous positive airway pressure (CPAP) or positive pressure ventilation (PPV).

**Results:**

Results are given as median (IQR) or percentages where appropriate. Suitable recordings were made in 50 infants (GA 27 (26–29) weeks), 17 received CPAP and 33 PPV. SpO_2_ increased rapidly in the first minute after FiO_2_ 1.0 and remained stable. The duration of FiO_2_ 1.0 tended to be shorter in the CPAP group than in the PPV group (CPAP vs. PPV: 65 (33–105) vs. 100 (40–280) s; p = 0.05), SpO_2_ >95% occurred more often in PPV group (53% vs. 69%) and lasted longer (70(40–95) vs. 120(50–202) s). In CPAP group, minute volume increased from 134 (76–265) mL/kg/min 1 minute before to 240 (157–370) mL/kg/min (p<0.01) 1 minute after start FiO_2_ 1.0 and remained stable at 2 minutes (252 (135–376) mL/kg/min; ns). The rate of rise to maximum tidal volume increased (from 13.8 (8.0–22.4) mL/kg/s to 18.2 (11.0–27.5) mL/kg/s; p<0.0001) to 18.8 (11.8–27.8) mL/kg/s; ns). In the PPV group respiratory rate increased from 0(0–4) to 9(0–20) at 1 minute (p<0.001) to 23 (0–34) breaths per minute at 2 minutes (p<0.01).

**Conclusion:**

In preterm infants at birth, a rapid increase in oxygenation, resulting from a transient increase to 100% oxygen might improve respiratory drive, but increases the risk for hyperoxia.

## Introduction

Hyperoxemia may lead to hyperoxia causing oxidative stress and tissue injury which should be avoided in infants at birth [1], [2]. Meta-analyses indicate that resuscitation of term infants at birth with air significantly reduced mortality compared with those resuscitated with fraction of inspired oxygen (FiO_2_) of 1.0 [1]–[6]. International resuscitation guidelines now recommend term infants should start in air [2], [7], [8]. Less clinical data are available for preterm infants, but guidelines now recommend to use oxygen judiciously during stabilization of preterm infants at birth [2], [7]–[9].

Since SpO_2_ percentiles were introduced [10] lower SpO_2_-targets in the first minutes after birth are accepted. However, hypoxia inhibits breathing movements in the fetus [11]. Although O_2_ sensitivity of infants changes in days-weeks after birth [12] and most preterm infants breathe at birth [13], [14], it is not known when the hypoxia-mediated switch from respiratory suppression to stimulation occurs. Possibly hypoxia immediately after birth will produce a weakened or absent respiratory drive as shown in preterm lambs [12]. In contrast, it has been shown in asphyxiated term infants [15] and animals [16] that applying 100% oxygen with no titration delayed the time of the first breath.

From 2008 until 2010, the local guidelines of the Royal Women's Hospital (Melbourne, Australia) and the Leiden University Medical Center (Leiden, the Netherlands) recommended starting in air and switching to FiO_2_ 1.0 if needed and then titrating down in preterm infants at birth. An oxygen saturation (SpO_2_) ≤70% at 5 minutes was used to increase FiO_2_ [10]. The immediate switch to 100% was a pragmatic choice, but immediate FiO_2_ reduction was advocated once the infant was stabilized.

Our aim was to investigate the change in SpO_2_ and respiratory drive in preterm infants right after birth in the delivery room after switching from air to FiO_2_ 1.0.

## Methods

The local institutional review boards (IRBs) of the Leiden University Medical Center (Commissie Medische Ethiek, Leids Universitair Medisch Centrum) and Royal Women's Hospital (the Human Research Ethics Committee, Royal Woman's Hospital) approved physiological- and video recordings at birth in the delivery room when respiratory support was necessary for research purposes. Written parental consent to use the recordings for research was obtained after birth. A retrospective study was performed in both hospitals with data collected between 2008 and 2010. During the period of data collection local guidelines recommended that support was started with air and switched to FiO_2_ 1.0 when: 1) cardiac massage was needed, 2) positive pressure ventilation (PPV) was administered for 1 minute and heart rate (HR) was <100 beats per minute (bpm) or 3) SpO_2_ <70% at 5 minutes. FiO_2_ was then titrated down as quickly as possible (when SpO_2_ >90%). Recordings were only made when the research team was available.

Respiratory support was delivered with a T-piece resuscitator (Neopuff, Fisher & Paykel, Wellington, New Zealand) and face mask. Local resuscitation guidelines recommended to start PPV (20–25/5 cmH_2_O) in preterm infants during apnea or HR<100 bpm. In breathing infants and HR>100 bpm, CPAP (5–6 cmH_2_O) is given. Changing pressures was left to the discretion of the caregiver.

The use of a respiratory monitor (Acutronic Medical Systems AG, Hirzel, Switzerland), a Masimo SET pulse oximeter (Masimo Radical, Masimo Corporation, Irvine CA, USA), an oxylog (Teledyne, Poway CA, USA) and Spectra program (Spectra, Grove Medical Limited, Hampton, UK) for physiological recordings has been described in detail in previous publications [13].

All recordings of infants born at <32 weeks gestation between 2008 and 2010 were reviewed. Using video and respiratory function monitoring other interventions were identified performed during the analyzed period. Infants receiving FiO_2_ 1.0 were identified and divided into two groups. This was based on the type of respiratory support they received around the time point FiO_2_ 1.0 was started: group 1) infants were breathing on continuous positive airway pressure (CPAP) and group 2) received positive pressure ventilation (PPV).

In all infants we recorded when FiO_2_ was increased to 1.0, for what reason(s) (e.g. low HR, low SpO_2_), duration and the downward titration rate of FiO_2_ 1.0. Furthermore we noted the increase in SpO_2_ duration of SpO_2_ >95%. We used SpO_2_ >95% as an indication for increased risk for hyperoxia.

In group 1 (CPAP group) the effect of FiO_2_ 1.0 on respiratory drive was investigated. To measure the change in respiratory effort, we analyzed the respiratory rate (RR), expired tidal volume (Vte), minute volume (MV) and the rate of rise to maximum tidal volume (mL/kg/second) during inspiration from 1 minute before until two minutes after starting FiO_2_ 1.0 (which served as a control period). To measure the maximum rate of tidal volume increase we used spontaneous breaths without mask leak (Vti = Vte).

In group 2 (PPV group) the tidal volumes and rate of rise will be influenced by the positive pressure ventilation given and we only analyzed RR of the spontaneous breaths from 1 minute before until two minutes after starting FiO_2_ 1.0. Breaths in between and coinciding with inflations were identified according to previous described methods [15]. In apneic infants, starting time of breathing was noted.

As changing of FiO_2_ can influence flow and volume measurements [17]–[19], the respiratory monitor was tested in vitro by delivering a constant tidal volume using a glass syringe and different gas conditions. The results were used to give the following corrections: at FiO_2_ 1.0, both inspired and expired tidal volumes were corrected by −6% when using cold dry gas and by −10% when heated gas was used.

Data are presented as mean (± SD) or median (IQR) where appropriate. Differences were analyzed with a paired samples t-test for parametric data or a Wilcoxon signed rank test for non-parametric data where appropriate using (SPSS for Windows, version 17.0.0, Chicago, IL, USA). A two-sided p-value <0.05 was considered statistically significant.

## Results

Data from 80 recorded infants were reviewed, 30 were excluded (no respiratory support (n = 10), no supplemental oxygen (n = 7), low quality recordings (n = 12) and 1 infant was born dead). Thus, 50 infants with GA 27 (26–29) weeks were analyzed (table 1); during the study window (1 minute before–2 minutes after start of oxygen) 17 breathed on CPAP (CPAP-group) and 33 received PPV (PPV-group).

**Table 1:** Baseline characteristics for preterm infants breathing on CPAP and infants receiving positive pressure ventilation when a FiO_2_ of 1.0 was started.

Characteristics	breathing on CPAP	Positive pressure ventilation	p-value
	N = 17	N = 33	
Gestational age, wk, mean (SD)	28.9 (1.5)	27.1 (2.1)	<0.01
Birth weight, g, mean (SD)	1073 (227)	993 (311)	<0.0001
Male Sex (%)	10 (60)	16 (49)	ns
Caesarean (%)	8 (47)	18 (54)	ns
Apgar at 1 min, median (IQR)	6 (5–7)	4 (2–6)	<0.05
Apgar at 5 min, median (IQR)	8 (8–8)	7 (6–8)	<0.05

The infants in the CPAP-group did not receive PPV during or after the study window. In both groups ventilation pressures were not increased during the study window. (pressures given: CPAP-group CPAP level 5.3 (4.6–5.9) cmH_2_O, PPV-group; PIP 21.6 (20.3–24.9) cmH_2_O and PEEP 4.2 (3.5–4.7) cmH_2_O) and no readjustments of mask position were observed. After the study window 1 infant of the CPAP-group was intubated, but reason was unclear. In the PPV-group, 5 infants were intubated after the study window for apnea and low SpO_2_ despite FiO_2_ 1.0. Cardiac massage was not provided to any infant.

### Supplemental oxygen

In the CPAP-group FiO_2_ 1.0 was started 300 (225–315) seconds after birth and was given for 65 (33–105) seconds. FiO_2_ was weaned in 20 (5–60) seconds to 21 (21–21) %. In all infants oxygen was started for low SpO_2_ and HR was >100 bpm.

In the PPV-group FiO_2_ 1.0 was started 180 (120–270) seconds after birth and was given for 100 (40–280) seconds. FiO_2_ was weaned in 25 (10–47) seconds to 21 (21–30)%.

### Oxygen saturation

**Figure 1:**
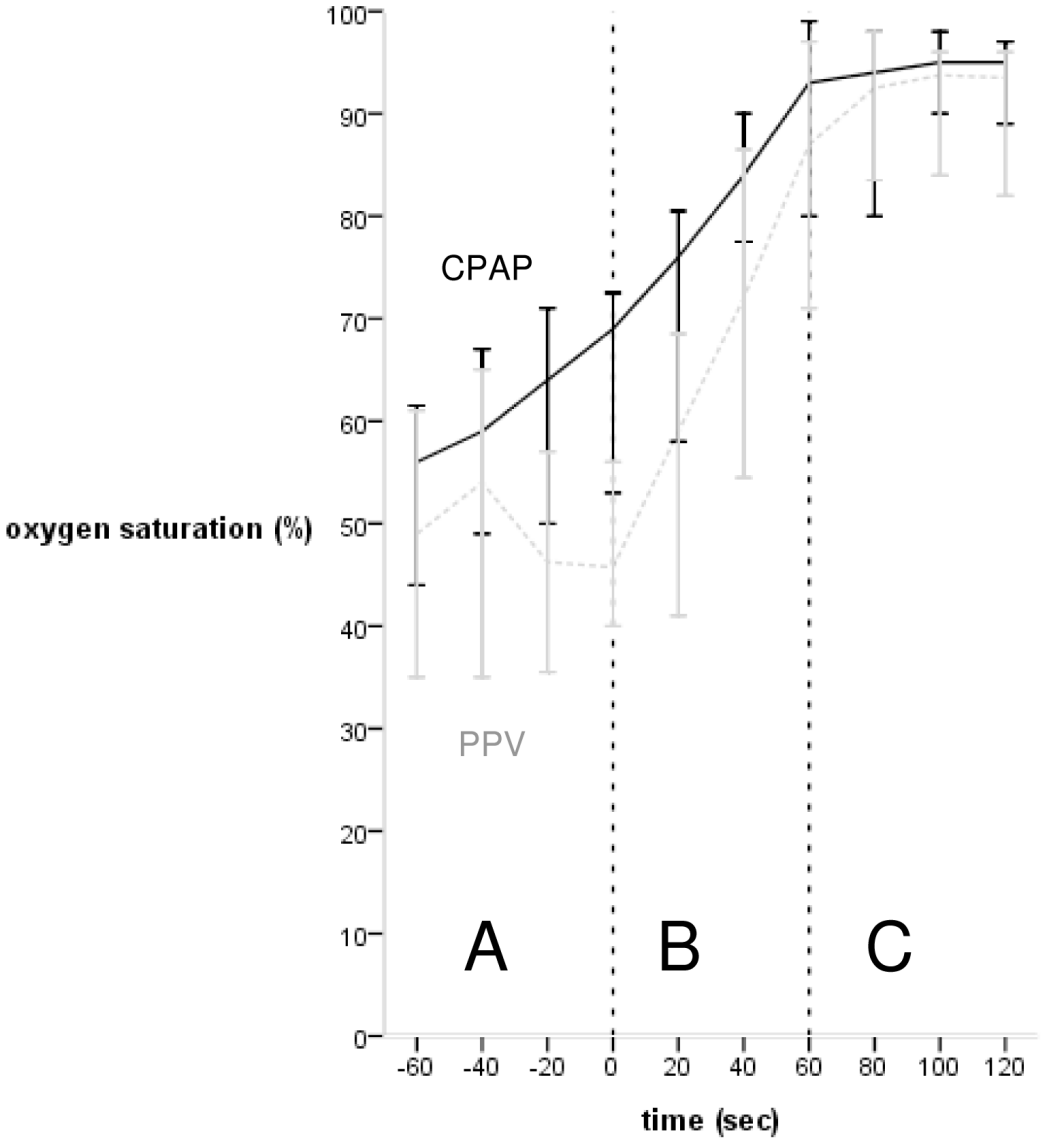
Oxygen saturation (%) of infants on CPAP and infants needing PPV in the minute before and 2 minutes after start of FiO_2_ 1.0. Black = CPAP-group, light grey = PPV-group, (A) minute before start FiO_2_ 1.0, (B) first minute after start FiO_2_ 1.0, (C) second minute after start FiO_2_ 1.0.

In all patients the fastest increase in SpO_2_ occurred in the first minute after starting oxygen (figure 1) (CPAP-group: from 62 (16)% to 87 (12)% at 1 minute after and to 93 (5)% at 2 minutes after starting oxygen, PPV-group: from 45 (19)% to 80 (24)% (p<0.001) after 1 minute and to 87 (19)% after 2 minutes (figure 1).

**Figure 2:**
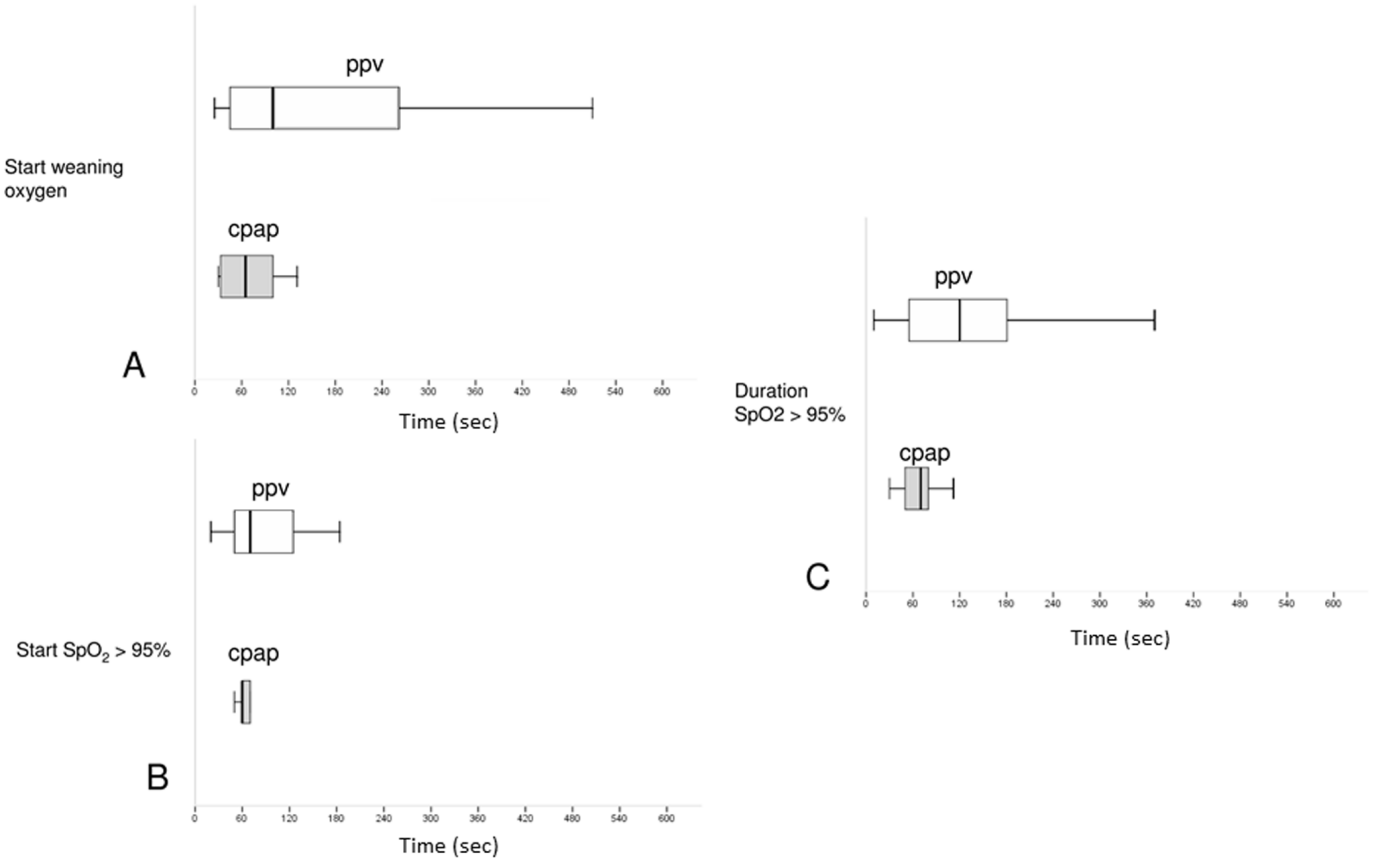
Box plots showing median (IQR) starting time of weaning FiO_2_ 1.0 (A), starting time of SpO2 >95%, (B) in seconds after FiO_2_ 1.0 is started and duration of SpO2 >95%, (C) in infants on CPAP and infants needing PPV. Grey = CPAP-group, white = PPV-group.

SpO_2_ >95% occurred in 9/17 (53%) infants of in group 1 and in 23/33 (69%) infants of group 2. The starting point and duration of SpO_2_ >95% are depicted in figure 2

### Changes on respiratory drive when breathing on CPAP

In the CPAP-group, increasing FiO_2_ to 1.0 increased RR from 30 (18–41) 1 min before to 35 (24–45) breaths per minute (ns) 1 minute after to 39 (31–44) breaths per minute (p<0.05) in the 2^nd^ minute.

Vte and MV increased significantly in 1^st^ minute after increasing FiO_2_ to 1.0 and remained stable in the 2^nd^ minute (Vte: from 4.9 (2.3–8.8) mL/kg to 6.7 (3.6–10.4) mL/kg (p<0.001) to 6.5 (3.7–10.2) mL/kg (ns); MV: from 134 (76–265) mL/kg/min to 240 (157–370) mL/kg/min (p<0.01) to 252 (135–376) mL/kg/min (ns)).

The rate of rise to maximum tidal volume increased from 13.8 (8.0–22.4) mL/kg/s in the minute before to 18.2 (11.0–27.5) mL/kg/s (p<0.0001) in the minute after increasing FiO_2_ to 1.0 and remained stable at 18.8 (11.8–27.8) mL/kg/s (ns) in the 2^nd^ minute.

### Changes on respiratory drive when receiving PPV

PPV was given in 23 apneic infants and in 10 infants for poor respiratory drive. Apneic infants started breathing 80 (50–180)s after FiO_2_ 1.0 and at that moment SpO_2_ was 87% (11) and HR 147 (19) bpm.

RR increased from 0 (0–4) 1 min before to 9 (0–20) breaths per minute (p<0.001) 1 minute after to 23 (0–34) breaths per minute (p<0.01) in the 2^nd^ minute after switching to FiO_2_ 1.0.

## Discussion

We investigated the influence of switching from air to FiO_2_ 1.0 on SpO_2_ and respiratory drive in preterm infants at birth. Most infants with SpO_2_ near the 10^th^ percentile had a good HR but FiO_2_ was increased to 1.0 because of low SpO_2_. After increasing FiO_2_ to 1.0, respiratory drive improved simultaneously with a rapid increase in SpO_2_. However, SpO_2_ >95% occurred in the majority of infants, especially in the infants who received PPV, which probably reflects the difficulty of simultaneously performing PPV and titrating oxygen. These observations suggest that targeting a higher percentile as currently recommended in international guidelines [7], [8] (25^th^–50^th^ percentile) might improve respiratory drive. A more stepwise increase in FiO_2_ and more diligence in reducing FiO_2_, for example when SpO_2_ >85%, could reduce the risk of hyperoxia.

We observed that preterm infants started to breathe more vigorously, as indicated by an increased rate and effort, once FiO_2_ was increased and SpO_2_ improved. Antenatally, hypoxia suppresses fetal breathing movements [11] whereas postnatally hypoxia stimulates breathing. The sensitivity increases during days-weeks after birth [12]. However, the mechanisms driving the large inspiratory efforts and controlling the switch to continuous breathing after birth are unknown, although increasing arterial PO_2_ may be involved [20]. We speculate that infants in our study, who failed resuscitation with air, respiratory support was insufficient to aerate the lung and supplemental oxygen was required to compensate. We suggest that the resulting increase in oxygenation increased drive from the respiratory center and respiratory effort, which increased lung aeration and FRC. This would explain why FiO_2_ 1.0 was only required for a short time and could be rapidly weaned allowing most infants to remain stable with little extra oxygen. Although our weaning rate was fast, studies comparing high versus moderate FiO_2_ levels in preterm infants found similar levels of FiO_2_ at 10 minutes [9], [21]–[23].

Experimental studies have shown that pulmonary vascular resistance at birth is related to ventilation onset and oxygen had little impact [24]–[26]. Also, Sobotka et al. found that increasing FiO_2_ to 1.0 in hypoxic lambs just after birth improved blood oxygenation, but had no effect on lung compliance and pulmonary blood flow [25]. This supports the hypothesis that increased oxygenation after FiO_2_ 1.0 is achieved by increasing the partial pressure gradient for oxygen diffusion compensating the ventilation perfusion mismatch due to low FRC [25].

The reported Vte increased in infants on CPAP could be explained by improved lung compliance. However, volume increase occurred right after increasing FiO_2_ and remained stable in the minute thereafter. Also, RR increase cannot be explained by improving compliance. Alternatively, increased pressures could have elevated Vte, but these remained unchanged. Increasing FiO_2_ increases gas density which can influence measurements [17]–[19]. However, after correction, tidal volumes remained significantly larger after FiO_2_ 1.0 and when considering the rate of rise is also increased it is more likely to be the infant's own effort.

Although studies showed it is feasible to support preterm infants at birth with a FiO_2_ of <1.0, most infants starting with low FiO_2_ levels needed an increased FiO_2_ (0.45–0.6) to reach target SpO_2_ levels [21], [22]. However, the different approaches make it difficult to compare these studies with our observational data reported in this study. We often observed SpO_2_ below target. Therefore, starting in air may not be the right approach. Although it is unclear how detrimental a short period of FiO_2_ 1.0 is at birth (1–2 minutes), more vigilance in preventing SpO_2_ levels >95% is needed [2].

In line with our recent report [13], we observed that oxygen use was not always according to the guidelines. Oxygen was given earlier or later than recommended. In the PPV-group a SpO_2_ >95% occurred more often and lasted longer, increasing the chances of hyperoxia. These observations may indicate the algorithm was difficult to follow. This will become even more difficult if separate SpO_2_ targets for each minute after birth are defined and may lead to a change in focus away from adequate ventilation. Adding an extra person to the resuscitation team could be helpful.

### Limitations

The retrospective nature of the study and the relative small sample size precludes any hard conclusion regarding respiratory drive and oxygenation. Recording respiratory parameters at birth is challenging, similar studies do not include large number of infants [27]–[31]. The infants included in this study is a sample of the preterm infants born in the hospitals and a selection bias could have occurred. However, the sample was randomly chosen as recordings were performed if the research team was available. The observed variation in starting time of FiO_2_ 1.0 complicates comparing with the respiratory drive of infants receiving air. Also, the observational nature of this study prevented us to have a control group of infants needing no support. However, we were interested in the effect of FiO_2_ 1.0 and measurements before starting oxygen served as a control period.

### Conclusions

We observed that during respiratory support of preterm infants switching from air to FiO_2_ 1.0 increased the risk for hyperoxia. No hard conclusions can be drawn, but our observations might suggest that respiratory drive increased after supplemental oxygen was given and oxygenation improved. The role of SpO_2_ levels in stimulating respiratory drive at birth merits further investigation.
